# Role of Arterial Blood Gas in Risk Stratification of Patients With Acute Exacerbation of Chronic Obstructive Pulmonary Disease in the Emergency Department: A Systematic Review

**DOI:** 10.7759/cureus.94369

**Published:** 2025-10-11

**Authors:** Iman Fatima, Jaipal Dass, Zakia Rauf Aslam, Muhammad Miraj Khan, Hassan Imtiaz, Havil Stephen Alexander Bakka, Muhammad Aqib Mazhar, Saif Abdulsattar, Areeba Zahid, Inam Rafiq

**Affiliations:** 1 General Medicine, King’s College Hospital, London, GBR; 2 Emergency, Walsall Healthcare National Health Service (NHS) Trust, Birmingham, GBR; 3 Accident and Emergency, Barking Havering and Redbridge University Hospital, London, GBR; 4 Care of the Elderly, Barking Havering and Redbridge University Hospital, London, GBR; 5 Trauma and Orthopaedics, University Hospitals Dorset, Poole, GBR; 6 Neurosurgery, Royal Sussex County Hospital, Brighton, GBR; 7 General Surgery, Northwick Park Hospital, Harrow, GBR; 8 Trauma and Orthopaedics, Manchester University National Health Service (NHS) Foundation Trust, Manchester, GBR; 9 Medicine, Faisalabad Medical University and Hospital, Faisalabad, PAK; 10 Internal Medicine, Dow University of Health Sciences, Civil Hospital Karachi, Karachi, PAK

**Keywords:** aecopd, arterial blood gas, copd, emergency department, hypercapnic respiratory failure, risk stratification

## Abstract

Acute exacerbations of chronic obstructive pulmonary disease are a leading cause of emergency department visits and hospital admissions, associated with high morbidity, mortality, and healthcare costs. Accurate risk stratification in the emergency department is vital for identifying patients at risk of deterioration and guiding decisions regarding intensive care unit admission or ventilatory support. Arterial blood gas analysis remains the gold standard for assessing respiratory failure, providing direct measurements of potential of hydrogen (pH), partial pressure of carbon dioxide in arterial blood (PCO₂), and partial pressure of oxygen in arterial blood (PaO₂), as well as the fraction of inspired oxygen (PaO₂/FiO₂). This systematic review, based on five eligible studies with a total sample size of 1,305 patients, found that arterial blood gas derangements such as acidosis, hypercapnia, and impaired oxygenation consistently predicted poor outcomes, including increased mortality and need for invasive ventilation. Compared to clinical scores and venous blood gas analysis, arterial blood gas offered more precise insights into ventilatory compromise and gas exchange abnormalities. These findings support arterial blood gas as an indispensable tool in the early risk stratification of acute exacerbation of chronic obstructive pulmonary disease. patients in the emergency department, complementing existing clinical models and improving prognostic accuracy. Larger multicenter studies are warranted to validate arterial blood gas-integrated prediction frameworks and optimize triage strategies in this high-risk population.

## Introduction and background

Chronic obstructive pulmonary disease (COPD) is a leading cause of morbidity and mortality worldwide, with acute exacerbations (AECOPD) accounting for a large proportion of emergency department (ED) visits and hospital admissions [1]. These episodes accelerate the decline in lung function, increase healthcare costs, and contribute to poor long-term outcomes. Effective early risk stratification in the ED is therefore essential to reduce mortality and optimize resource allocation. Several clinical tools, such as CRB-65: confusion, respiratory rate ≥30/min, blood pressure (systolic <90 mmHg or diastolic ≤60 mmHg), and age ≥65 years [2].CURB-65: confusion, urea >7 mmol/L, respiratory rate ≥30/min, blood pressure (systolic <90 mmHg or diastolic ≤60 mmHg), age ≥65 years [3]. BAP-65: blood urea nitrogen ≥25 mg/dL, altered mental status, pulse ≥109/min, age ≥65 years, and the National Early Warning Score (NEWS) have been employed to guide triage decisions in AECOPD [4]. While these scores are useful in assessing general disease severity, they were not designed specifically for COPD exacerbations and may not accurately reflect respiratory failure when ABG data are available.

While these scores are useful, they were not originally designed for COPD-specific exacerbations and may lack sensitivity in detecting early ventilatory compromise. This limitation highlights the need for objective physiological markers that directly reflect respiratory status. Arterial blood gas (ABG) analysis remains the gold standard in assessing respiratory failure during AECOPD. Key parameters, including potential of hydrogen (pH) (blood acidity/alkalinity), PaCO₂ (partial pressure of arterial carbon dioxide), and PaO₂ (partial pressure of arterial oxygen provide vital information on the severity of hypoxemia and hypercapnia, while the PaO₂/FiO₂ ratio helps quantify gas exchange efficiency [5].

Importantly, abnormalities such as respiratory acidosis and significant CO₂ retention are directly linked to the need for ventilatory support and adverse outcomes. Despite its widespread use, the prognostic role of ABG in AECOPD has been variably reported. Some studies emphasize its predictive value for mortality and intensive care unit (ICU) admission, while others suggest that ABG should be interpreted in combination with clinical scores. This systematic review aims to evaluate the role of ABG in risk stratification of patients presenting with AECOPD to the ED, focusing on its prognostic significance and integration with existing clinical tools.

## Review

Materials and methods

Search Strategy

A systematic search was performed from January 2010 to June 2025 across PubMed, Embase, Scopus, and the Cochrane Library. Keywords and MeSH terms used included “COPD,” “acute exacerbation,” “arterial blood gas,” “ABG,” “risk stratification,” “hypercapnic respiratory failure,” and “emergency department.” Boolean operators such as “AND” and “OR” were applied to maximize sensitivity while reducing irrelevant results. Filters were applied to exclude pediatric populations, non-English studies, and publications without available abstracts. Reference lists of included articles were also screened to identify additional eligible studies. The search and selection process followed PRISMA (preferred reporting items for systematic reviews and meta-analyses) guidelines, ensuring transparency and reproducibility in study identification and inclusion [6].

Eligibility Criteria

Eligibility was determined using the PICO framework. Population (P): Adult patients diagnosed with AECOPD presenting to the ED or ICU [7]. Intervention/Exposure (I): Arterial blood gas analysis parameters, including pH, PaCO₂, PaO₂, and PaO₂/FiO₂ ratio. Comparator (C): Clinical scoring systems (e.g., CRB-65, CURB-65, BAP-65), venous blood gas (VBG), or stratification by severity categories. Outcomes (O): The primary outcome was in-hospital mortality, while secondary outcomes included ICU admission, need for invasive or noninvasive ventilation, and long-term mortality. Studies were included if they reported ABG findings linked to clinical outcomes. Exclusion criteria encompassed pediatric patients, case reports, reviews, animal studies, and studies without outcome analysis. Only observational cohort studies were included. Randomized controlled trials were excluded. Outcomes were categorized as short-term (in-hospital) and long-term (≥90 days) mortality.

Study Selection

Two independent reviewers screened all retrieved records. Titles and abstracts were first examined to eliminate irrelevant studies, followed by a full-text review to confirm eligibility. Discrepancies in study inclusion were resolved through discussion and consensus. Initial screening eliminated duplicates, animal studies, case reports, and editorials. Only studies with original data linking ABG results to clinical outcomes were retained. This multistage screening ensured that the final selection represented high-quality evidence directly relevant to the role of ABG in AECOPD risk stratification.

Data Extraction

Data were systematically extracted using a pre-designed form. Information collected included study design, author and year, sample size, patient population, ABG parameters measured, comparator tools, and outcomes reported. Where available, additional data such as comorbidity burden, adjunct laboratory findings, and length of stay were also noted. Extraction was performed independently by two reviewers to minimize bias, and cross-checking was conducted for accuracy. Extracted data were then synthesized into summary tables for structured comparison across studies.

Risk of Bias Assessment

Risk of bias in included studies was evaluated using the Newcastle-Ottawa Scale (NOS), a validated tool for observational research [8]. All included studies were observational; therefore, the Newcastle-Ottawa Scale (NOS) was the appropriate tool for assessing risk of bias. Each study was assessed across three domains: selection of participants, comparability of study groups, and ascertainment of outcomes. Scores were used to classify studies as having low, moderate, or high risk of bias. Two reviewers independently performed the assessment, and discrepancies were resolved by consensus. This process ensured methodological rigor and allowed for cautious interpretation of results in light of study limitations.

Data Synthesis

Due to heterogeneity in study design, outcome measures, and populations, a narrative synthesis was performed rather than a meta-analysis. Results from individual studies were compared and organized according to major ABG parameters: pH, PaCO₂, PaO₂, and PaO₂/FiO₂ ratio. Trends across studies were highlighted, particularly in relation to mortality, ventilation needs, and ICU admission. Findings were then contextualized with existing clinical scoring systems to evaluate the incremental value of ABG in AECOPD triage. This structured approach allowed meaningful synthesis despite differences in methodology.

Ethical Considerations

This study is a systematic review of previously published data and did not involve human participants or animal subjects; therefore, ethical approval was not required. The review was not prospectively registered on PROSPERO or any other registry.

Results

Study Selection Process

**Figure 1:**
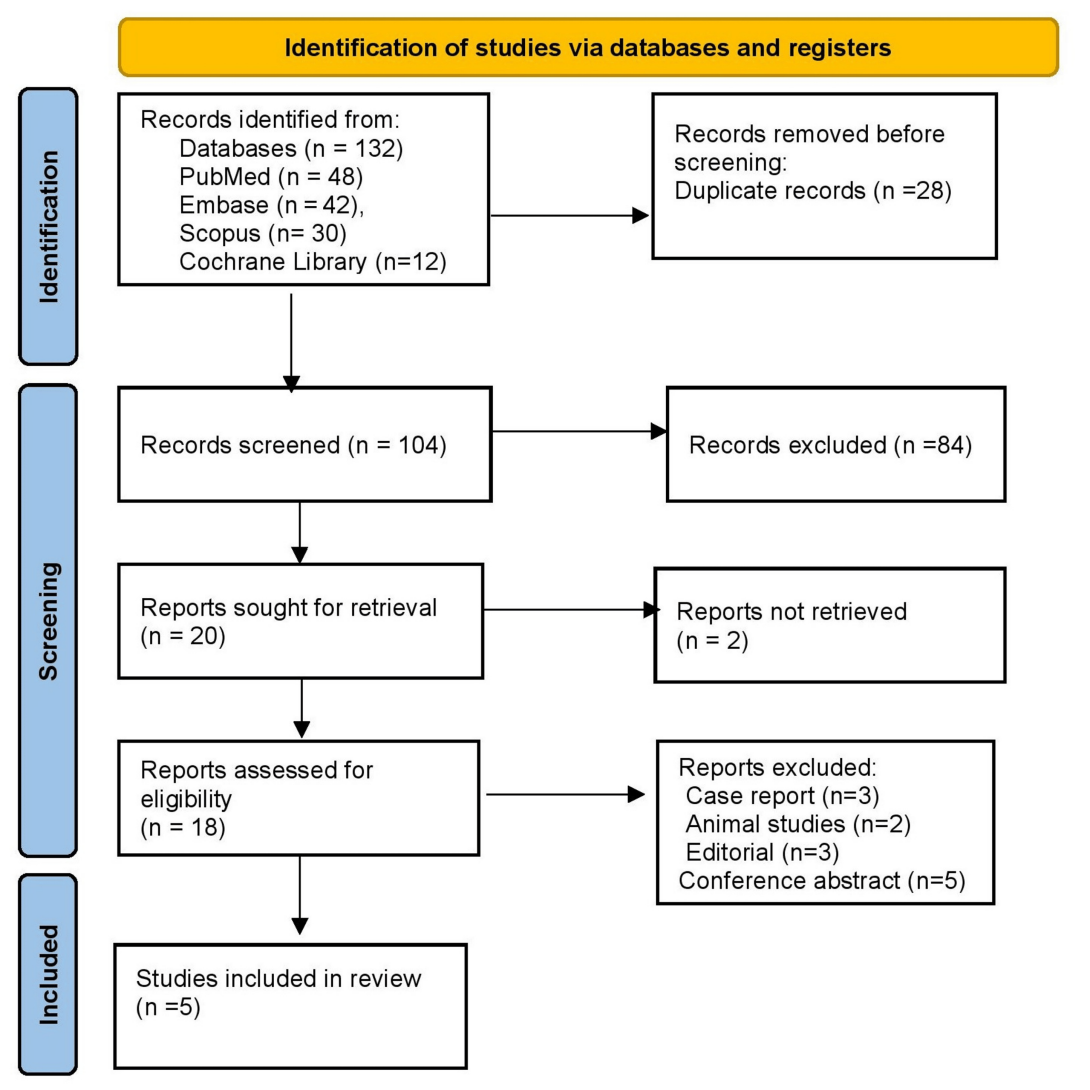
PRISMA 2020 flow diagram

Figure 1 shows a total of 132 records were identified across databases: PubMed (48), Embase (42), Scopus (30), and Cochrane Library (12). After the removal of 28 duplicates, 104 records remained for screening. Of these, 84 were excluded based on titles and abstracts, leaving 20 full-text articles for assessment. Two studies could not be retrieved in full text, and 18 were assessed for eligibility. Exclusions included case reports (3), animal studies (2), editorials (3), and conference abstracts (5). Finally, five studies met the inclusion criteria and were included in the review. The PRISMA flow diagram summarizes this process, demonstrating the systematic approach to study selection.

Characteristics of the Selected Studies

Table 1 summarizes major studies assessing the role of ABG in AECOPD risk stratification. Chen et al. (2021) showed that low pH and elevated PaCO₂ predicted mortality and ICU admission, outperforming CRB-65 and CURB-65 scores [9]. McKeever et al. (2016) reported that while venous blood gas could approximate pH and PaCO₂, only ABG reliably measured PaO₂ for oxygenation assessment [10]. Christensen et al. (2008) found that severe ABG derangements before intubation correlated with poor long-term survival in ventilated COPD patients [11]. Kumar et al. (2022) highlighted that acidosis and hypercapnia, combined with metabolic markers, predicted in-hospital mortality [12]. Akbaş et al. (2022) demonstrated that low PaO₂/FiO₂ ratios and acidosis strongly predicted ICU and 90-day mortality [13]. Together, these studies confirm ABG as a cornerstone tool for identifying high-risk patients and guiding escalation of care. 

**Table 1:** Characteristics of the selected studies ABG: Arterial Blood Gas, AECOPD: Acute Exacerbation of Chronic Obstructive Pulmonary Disease, ED: Emergency Department, ICU: Intensive Care Unit, VBG: Venous Blood Gas, pH: Potential of Hydrogen, PaCO₂: Partial Pressure of Arterial Carbon Dioxide, PaO₂: Partial Pressure of Arterial Oxygen, PaO₂/FiO₂: Ratio of arterial oxygen partial pressure to inspired oxygen fraction, MV: Mechanical Ventilation, CRB-65 / CURB-65 / BAP-65 / NEWS: Clinical risk stratification scores

Author & Year	Population (P)	Exposure / Condition (I)	Comparator (C)	Outcomes (O)	ABG Interpretation	Physiology	Importance
Lan Chen et al. (2021) [9]	601 ED patients with AECOPD and hypercapnic respiratory failure	ABG: PaCO₂, pH, PaO₂ at admission; vitals and labs	Compared across CRB-65, CURB-65, BAP-65, NEWS scores	In-hospital mortality, invasive ventilation, ICU admission	pH <7.35 and PaCO₂ >50 mmHg indicate acute ventilatory failure	Hypercapnia reflects alveolar hypoventilation; acidosis from CO₂ retention	Identifies patients at high risk for ICU admission or death
McKeever TM et al. (2016) [10]	234 AECOPD patients in UK ED	ABG vs VBG (pH, PaCO₂, HCO₃⁻, PaO₂)	ABG compared with venous samples	Severity of exacerbation; ventilation decisions	VBG can approximate pH and PaCO₂ but not PaO₂	Gas exchange assessment is essential; only ABG measures oxygenation accurately	Guides when ABG is necessary in ED triage
Christensen S et al. (2008) [11]	230 COPD patients treated with invasive mechanical ventilation	ABG immediately before intubation (PaCO₂, PaO₂, pH)	Stratified by ABG derangement and comorbidity	90-day and 1-year mortality	Severe PaCO₂ elevation and acidosis signal decompensation	Reflects end-stage ventilatory failure with impaired alveolar ventilation	Prognosis worsens as oxygenation ratio (PaO₂/FiO₂) declines
Kumar et al. 2022, [12]	140 AECOPD patients admitted with severe exacerbation (India)	ABG: pH, PaCO₂, PaO₂; also sodium, sugar, SGPT	High vs low cut-offs	In-hospital mortality	Low pH and high PaCO₂ predict mortality	CO₂ retention leads to acidosis, worsened by electrolyte imbalance	Highlights ABG’s role in conjunction with metabolic markers
Akbaş T et al. (2022) [13]	100 ICU COPD patients with acute hypercapnic respiratory failure	ABG: PaO₂/FiO₂, PaCO₂, pH	Survivors vs. non-survivors	ICU and 90-day mortality	Low PaO₂/FiO₂ and acidosis strongly predict poor outcome	Impaired oxygen diffusion and ventilatory compromise	Reinforces ABG as critical for risk stratification in ICU COPD patients

Risk of Bias Assessment

Table 2 summarizes the risk of bias assessment using the Newcastle-Ottawa Scale (NOS). Chen et al. (2021) was rated moderate due to its single-center design despite a large cohort [9]. McKeever et al. (2016) showed low-moderate risk, with reliable measurements but some possible confounding [10]. Christensen et al. (2008) was moderate, as ABG data were clear, but long-term adjustment was limited [11]. Kumar et al. (2022) also had a moderate risk, with well-defined inclusion but a small sample and potential selection bias [12]. Akbaş et al. (2022) was moderate, with clear ABG assessment but incomplete confounder adjustment and ICU-only focus [13]. Overall, most studies were of moderate quality, requiring cautious interpretation. 

**Table 2:** Risk of bias assessment NOS: Newcastle–Ottawa Scale, ABG: Arterial Blood Gas, AECOPD: Acute Exacerbation of Chronic Obstructive Pulmonary Disease, ED: Emergency Department, ICU: Intensive Care Unit, MV: Mechanical Ventilation

Authors & Year	Study Design	Risk of Bias Tool	Risk of Bias Rating	Justification
Lan Chen et al. (2021) [9]	Prospective cohort (ED AECOPD patients)	Newcastle–Ottawa Scale (NOS)	Moderate	Single center but large sample; outcomes clearly defined; residual confounding possible
McKeever TM et al. (2016) [10]	Prospective observational (ED ABG vs VBG)	Newcastle–Ottawa Scale (NOS)	Low–Moderate	Good exposure/outcome measurement; some confounding possible; correlation study
Christensen S et al. (2008) [11]	Cohort (COPD patients on invasive MV)	Newcastle–Ottawa Scale (NOS)	Moderate	Older cohort; ABG measures clear; long-term outcomes variably adjusted
Kumar et al. (2022) [12]	Retrospective cohort (AECOPD, India)	Newcastle–Ottawa Scale (NOS)	Moderate	Inclusion criteria well defined; small sample size; potential selection bias
Akbaş T et al. (2022) [13]	Retrospective cohort (ICU COPD with hypercapnic RF)	Newcastle–Ottawa Scale (NOS)	Moderate	ABG exposure clear; adjustment for confounders incomplete; ICU-based sample only

Discussion

The findings of this review highlight the essential role of arterial blood gas (ABG) analysis in the risk stratification of patients presenting with acute exacerbation of chronic obstructive pulmonary disease (AECOPD) to the emergency department. ABG provides direct insights into acid-base balance and ventilatory status, parameters that clinical scoring systems such as CRB-65 (confusion, respiratory rate, blood pressure, age ≥65 years), CURB-65 (confusion, urea, respiratory rate, blood pressure, age ≥65 years), BAP-65 (blood urea nitrogen, altered mental status, pulse ≥109/min, age ≥65 years), and NEWS (National Early Warning Score) do not fully capture [14]. While clinical scores provide a general estimate of disease severity, they do not capture ventilatory failure. ABG parameters, such as particularly pH and PaCO₂, demonstrate higher sensitivity for predicting the need for ventilatory support and ICU admission, offering incremental prognostic value when combined with these scores. Derangements such as acidosis (pH <7.35), hypercapnia (partial pressure of carbon dioxide in arterial blood [PaCO₂] >50 mmHg), and impaired oxygenation (low ratio of partial pressure of oxygen in arterial blood [PaO₂] to fraction of inspired oxygen [FiO₂]) serve as early markers of acute ventilatory failure and are strongly associated with mortality, intensive care unit (ICU) admission, and the need for invasive ventilation. Thus, ABG functions not only as a diagnostic tool but also as a prognostic marker guiding timely escalation of care [15]. In clinical practice, criteria such as the presence of respiratory acidosis, significant carbon dioxide retention, or declining PaO₂/FiO₂ ratios are often used to determine the need for noninvasive or invasive ventilatory support.

Although venous blood gas (VBG) can approximate pH and PaCO₂, it cannot accurately assess oxygenation (PaO₂). Therefore, ABG remains the preferred method for evaluating both ventilatory and oxygenation abnormalities in AECOPD. VBGs can approximate pH and PaCO₂, but they fail to evaluate oxygenation; ABGs offer a comprehensive assessment of gas exchange abnormalities. This makes it indispensable for emergency physicians when prioritizing patients for ICU admission or advanced respiratory interventions, particularly in those with rapidly deteriorating status. Evidence from multiple studies reinforces this prognostic utility. Chen et al. [9] and Kumar et al. [12] demonstrated that low pH and high PaCO₂ independently predicted in-hospital mortality, while McKeever et al. [10] confirmed that ABG was superior to venous samples in assessing oxygenation. Christensen et al. [11] and Akbaş et al. [13] further established that ABG derangements, particularly reduced PaO₂/FiO₂ ratios and severe acidosis, strongly correlated with ICU and long-term mortality in ventilated COPD patients. Collectively, these findings affirm that ABG is a cornerstone for evidence-based risk stratification in AECOPD. Despite its diagnostic value, ABG analysis has inherent limitations, including timing variability relative to symptom onset, dependence on concurrent oxygen therapy, and procedural invasiveness that may cause patient discomfort. These factors can affect consistency and interpretation across studies. Inconsistent ABG timing may underestimate its prognostic value if measured after stabilization. Small sample sizes reduced statistical power, and variable inclusion criteria may have contributed to heterogeneity among outcomes. Among these, variability in ABG timing appears most critical, as measurements taken after clinical stabilization may underestimate true prognostic potential.

Rendering Review

Despite these consistent findings, several limitations must be acknowledged. First, the majority of included studies were single-center and observational in nature, which introduces risks of selection bias and limits external validity. Second, patient populations varied significantly in terms of baseline severity of COPD, comorbidities, and treatment protocols, creating heterogeneity that complicates pooled interpretation. Third, most studies did not adequately adjust for confounders such as prior hospitalizations, pharmacologic therapies (e.g., corticosteroids, bronchodilators), or baseline functional status, all of which may influence outcomes. Fourth, the timing and frequency of ABG sampling were inconsistent across studies, raising concerns about the comparability of results. Additionally, sample sizes were relatively small in several studies, reducing statistical power. Finally, publication bias toward positive findings cannot be excluded, particularly since negative or inconclusive studies are less frequently reported.

To strengthen the evidence base, future research should focus on large, multicenter prospective cohorts with standardized ABG measurement protocols, rigorous adjustment for confounders, and integration of ABG findings with validated clinical scoring systems. Such studies would help develop robust COPD-specific prognostic models to guide emergency department triage and critical care decisions.

## Conclusions

This systematic review highlights the critical role of arterial blood gas analysis in risk stratification of patients with acute exacerbation of chronic obstructive pulmonary disease presenting to the emergency department. Derangements such as low pH, elevated partial pressure of carbon dioxide in arterial blood (PaCO₂), and reduced PaO₂/FiO₂ ratio (partial pressure of oxygen in arterial blood/fraction of inspired oxygen) consistently predict poor outcomes, including mortality, intensive care unit admission, and need for invasive ventilation. While clinical scores like CRB-65, CURB-65, and BAP-65 offer useful frameworks, they fail to capture the full extent of ventilatory and gas exchange abnormalities. Arterial blood gas complements these tools by providing direct physiologic insights, enhancing prognostic accuracy, and guiding timely escalation of care. The current evidence, though promising, is limited by heterogeneity, small sample sizes, and predominantly observational study designs. Future large-scale, multicenter studies are needed to validate ABG-based criteria and integrate them with established clinical scores to create COPD-specific risk prediction models that improve triage and patient outcomes. Arterial blood gas analysis remains a vital tool for early risk stratification in AECOPD. Derangements in pH, PaCO₂, and PaO₂/FiO₂ reliably predict poor outcomes and complement clinical scores to improve prognostic accuracy. Future multicenter studies are needed to integrate ABG data into standardized COPD-specific prediction models.
